# Supplementary material for: Application of hydrophilic polymers for the preparation of tadalafil solid dispersions: micromeritics properties, release and erectile dysfunction studies in male rats
Source: PeerJ. 2022 May 26;10:e13482. doi: 10.7717/peerj.13482 (PMC9148559; doi:10.7717/peerj.13482)
Supplement: Supplemental Information 2 [file peerj-10-13482-s002.docx]

**Release Kinetic studies**

To analyze the mechanism of the drug, release the dissolution was within model-dependent kinetic equations. The percentage drug release values were plotted against time in zero-order kinetics. Log percentage drug release was plotted against the time (min) for first-order kinetics. The percentage of drug release was plotted against the square root time for the Higuchi release model. The cubic root of the initial drug concentration was subtracted by the cube root of the percentage drug remaining against the time (min) profile used to calculate the Hixson-Crowell model. The log value of percentage drug dissolved is plotted against log time for the Korsmeyer-Peppas model.

| **Zero order** | TK188 | TK31 | TK64 |
| --- | --- | --- | --- |
| R^2^ | 0.762219 | 0.840893 | 0.89489 |
| Slope | 0.71705 | 0.61058 | 0.600417 |
| Intercept | 23.95055 | 31.98065 | 23.99808 |
| **First order** |  |  |  |
| R^2^ | -0.92231 | -0.96021 | -0.94901 |
| Slope | -0.01383 | -0.01749 | -0.01797 |
| Intercept | 1.926299 | 1.976462 | 2.066954 |
| Higuchi |  |  |  |
| R^2^ | 0.786213 | 0.954925 | 0.982064 |
| Slope | 8.569958 | 8.03413 | 7.634698 |
| Intercept | 8.036078 | 14.24347 | 8.065878 |
| **Korsmeyer-pappas** |  | **HIGHEST** | **HIGHEST** |
| R^2^ | 0.219815 | 0.977803 | 0.995083 |
| n | 0.328308 | 0.313319 | 0.369285 |
| Intercept | 1.064473 | 1.342268 | 1.182061 |
| **Hixon Crowell** |  |  |  |
| R^2^ | 0.87752 | 0.940188 | 0.955764 |
| slope | 0.02732 | 0.019587 | 0.016414 |
| Intercept | -2.11279 | -1.93083 | -2.0973 |

**Sexual behavior parameters- measurement**

**Table 1: Effect of TK188 on the mount Latency (ML), mount frequency (MF), intromission latency (IL) and intromission frequency (IF) of male rats.**

|  | **ML (Secs)** | | |
| --- | --- | --- | --- |
| **Rats** | **NC** | **TDL-STD** | **TK188** |
| R1 | 119.34 | 45.23 | 49.29 |
| R2 | 117.85 | 45.12 | 46.32 |
| R3 | 127.17 | 46.23 | 47.32 |
| R4 | 123.2 | 47.62 | 49 |
| R5 | 125.21 | 45.81 | 51.56 |
| R6 | 124.3 | 44.23 | 51.26 |
| **Mean** | **122.85** | **45.71** | **49.13** |
| **SD** | **3.41** | **1.10** | **1.98** |

|  | **MF** | | |
| --- | --- | --- | --- |
| **Rats** | **NC** | **TDL-STD** | **TK188** |
| R1 | 4.24 | 13.53 | 12.08 |
| R2 | 4.24 | 13.09 | 12.02 |
| R3 | 3.41 | 13.84 | 12.98 |
| R4 | 4.76 | 13.59 | 12 |
| R5 | 4.39 | 13.29 | 11.38 |
| R6 | 4.62 | 14.21 | 12.9 |
| **Mean** | **4.28** | **13.59** | **12.23** |
| **SD** | **0.45** | **0.38** | **0.58** |

|  | **IL (secs)** | | |
| --- | --- | --- | --- |
| **Rats** | **NC** | **TDL-STD** | **TK188** |
| R1 | 245.15 | 87.61 | 92.5 |
| R2 | 239.21 | 83.95 | 90.71 |
| R3 | 265.3 | 85.39 | 97.66 |
| R4 | 234.98 | 89.32 | 91.2 |
| R5 | 240.35 | 93.24 | 86.43 |
| R6 | 249.38 | 88.33 | 88.67 |
| **Mean** | **245.73** | **87.97** | **91.20** |
| **SD** | **10.30** | **3.09** | **3.64** |

|  | **IF** | | |
| --- | --- | --- | --- |
| **Rats** | **NC** | **TDL-STD** | **TK188** |
| R1 | 2.52 | 11.27 | 9.94 |
| R2 | 2.26 | 11.45 | 9.56 |
| R3 | 2.64 | 11.04 | 10.42 |
| R4 | 2.34 | 11.45 | 9.83 |
| R5 | 2.22 | 11.36 | 9.96 |
| R6 | 2.54 | 11.05 | 9.93 |
| **Mean** | **2.42** | **11.27** | **9.94** |
| **SD** | **0.16** | **0.18** | **0.27** |

Values are expressed as mean ± S.E.M., n =6 rats/group.

* indicates significance compared to NC group at p< 0.05.

**ǂ** indicates significance compared to TDL-STD group at p< 0.05.

**Table 2: Effect of TK188 on the ejaculation latency in 1st series (EL-1), post ejaculatory interval (PEI) and ejaculation latency in 2nd series (EL-2) of male rats.**

|  | **EL-1 (secs)** | | |
| --- | --- | --- | --- |
| **Rats** | **NC** | **TDL-STD** | **TK188** |
| R1 | 376.62 | 487.82 | 478.51 |
| R2 | 384.28 | 483.59 | 463.71 |
| R3 | 387.3 | 495.86 | 465.45 |
| R4 | 377.54 | 510.32 | 468.4 |
| R5 | 370.47 | 484.39 | 459.46 |
| R6 | 369.84 | 512.43 | 475.57 |
| **Mean** | **377.68** | **495.74** | **468.52** |
| **SD** | **6.75** | **12.29** | **6.93** |

|  | **PEI** | | |
| --- | --- | --- | --- |
| **Rats** | **NC** | **TDL-STD** | **TK188** |
| R1 | 494.28 | 335.82 | 370.46 |
| R2 | 496.64 | 331.34 | 360.17 |
| R3 | 512.43 | 321.53 | 354.94 |
| R4 | 493.57 | 327.45 | 379.42 |
| R5 | 476.38 | 348.29 | 366.73 |
| R6 | 506.45 | 350.51 | 365.47 |
| **Mean** | **496.63** | **335.82** | **366.20** |
| **SD** | **11.84** | **11.00** | **8.06** |

|  | **EL-2 (secs)** | | |
| --- | --- | --- | --- |
| **Rats** | **NC** | **TDL-STD** | **TK188** |
| R1 | 402.42 | 482.32 | 453.54 |
| R2 | 392.41 | 496.22 | 473.81 |
| R3 | 395.83 | 488.56 | 455.59 |
| R4 | 388.4 | 474.34 | 447.49 |
| R5 | 358.92 | 480.11 | 459.61 |
| R6 | 434.56 | 473.4 | 443.16 |
| **Mean** | **395.42** | **482.49** | **455.53** |
| **SD** | **23.24** | **8.31** | **10.20** |

Values are expressed as mean ± S.E.M., n =6 rats/group.

* indicates significance compared to NC group at p< 0.05.

**ǂ** indicates significance compared to TDL-STD group at p< 0.05.

**
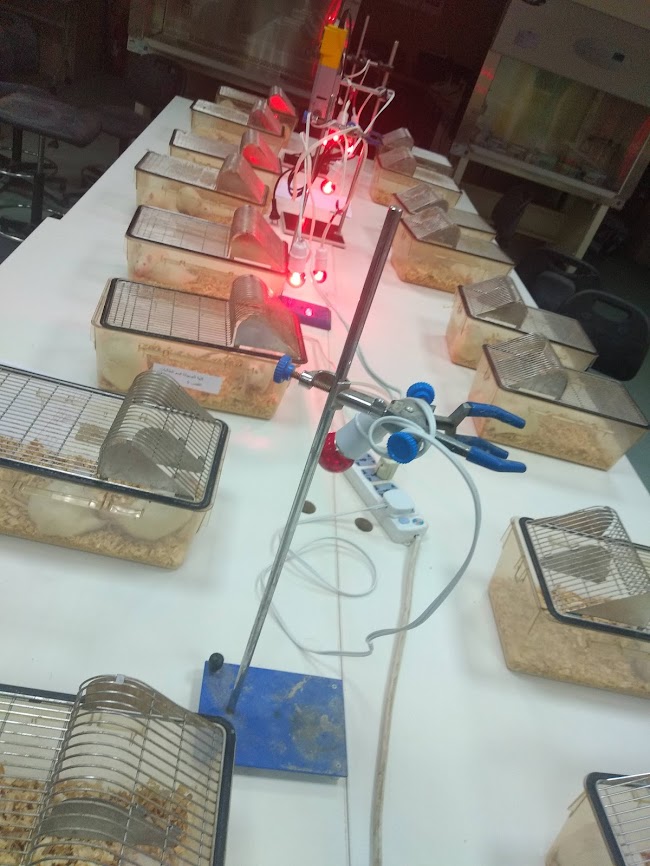
**

**
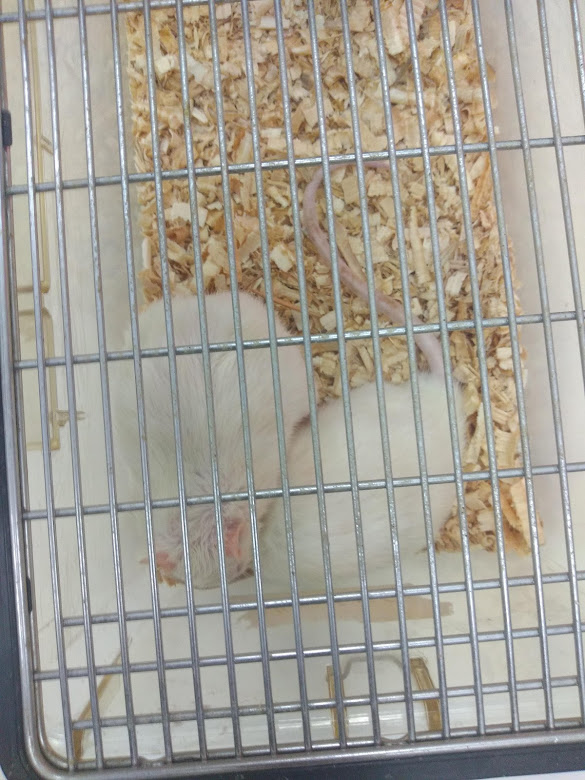
**
